# Comparison of localized retinal nerve fiber layer defects in highly myopic, myopic, and non-myopic patients with normal-tension glaucoma: a retrospective cross-sectional study

**DOI:** 10.1186/1471-2415-13-67

**Published:** 2013-11-05

**Authors:** Joon Mo Kim, Ki Ho Park, Seon Jeong Kim, Hyo Ju Jang, Eun Noh, Mi Jeung Kim, Tae Woo Kim, Dong Myung Kim, Joseph Caprioli

**Affiliations:** 1 Department of Ophthalmology, Sungkyunkwan University School of Medicine, Kangbuk Samsung Hospital, Seoul, South Korea; 2 Department of Ophthalmology, Seoul National University College of Medicine, Seoul National University Hospital, 28 Yongon-dong, Chongno-gu, Seoul 110-744, South Korea; 3 Seoul National University College of Medicine, Seoul, South Korea; 4 Department of Ophthalmology, Seoul National University Bundang Hospital, Seoul National University College of Medicine, Seongnam, South Korea; 5 Department of Ophthalmology, Jules Stein Eye Institute, University of California Los Angeles School of Medicine, Los Angeles, CA, USA

**Keywords:** Myopia, RNFL defect, Normal-tension glaucoma, Refractive error

## Abstract

**Background:**

The purpose of this study was to evaluate the relationship between patterns of localized retinal nerve fiber layer (RNFL) defects and the degree of myopia in patients with normal-tension glaucoma (NTG).

**Methods:**

We retrospectively reviewed medical records of patients with high myopia (42 eyes; spherical equivalent [SE] < −6.0 diopters [D]), low to moderate myopia (93 eyes; SE −6.0D ~ and −0.5D), and emmetropia (65 eyes; SE −0.5D ~ +0.5D), all of which were diagnosed as having NTG with localized RNFL defects. On RNFL photographs, the proximity of the RNFL defect to the center of the fovea (angle I) and the sum of the angular width of the defects (angle II) were determined. The patterns of localized RNFL defects were then compared with respect to differences in angles I and II.

**Results:**

Angle I was significantly smaller in the high myopia group than in the low to moderate myopia group (p = 0.028) and the emmetropia group (p = 0.044), while angle II was significantly larger in the high myopia group compared with the low to moderate myopia group and the emmetropia group (p < 0.001, p = 0.007).

**Conclusions:**

Among subjects with NTG, localized RNFL defects are wider and closer to the fovea in eyes with high myopia than those with low to moderate myopia or emmetropia.

## Background

Myopia is a serious health problem in many countries, and the incidence of high myopia appears to be rising in Asia and other parts of the world. In East Asian countries, the prevalence of myopia is high, and the number of new cases and the severity of the condition continue to increase [1]. An association between high myopia and glaucoma has been reported, with an increased prevalence of myopia in patients with ocular hypertension, primary open-angle glaucoma (POAG), and normal-tension glaucoma [2-4].

Retinal nerve fiber layer (RNFL) evaluation for detecting glaucomatous optic nerve damage has been increasingly used to detect damage. Numerous studies have confirmed the sensitivity of RNFL measurement in detecting glaucoma as well as the relationship between the extent of RNFL damage and the severity of functional deficits measured with perimetry [5-7]. Imaging devices are commonly used to detect glaucomatous RNFL damage [5-8]. Recently, some groups reported that the values measured by imaging devices could vary in myopic eyes [8,9]. A visual field (VF) test also has limitations in detecting glaucoma, especially in cases of high myopia due to myopic degeneration.

We have developed a quantitative method for analyzing localized RNFL defects with RNFL photography [10]. This method allows us to determine the location and width of the defects. In this study, we compare RNFL defects in highly myopic, low to moderately myopic, and non-myopic eyes using this method to investigate the relationship between myopia and RNFL defects in NTG patients.

## Methods

This was a retrospective clinical study. All subjects were diagnosed with NTG in the glaucoma clinic at Seoul National University Hospital between 2006 and 2007. A retrospective review of medical records was conducted of all newly diagnosed normal-tension glaucoma patients during the study period.

All patients received a complete ophthalmic examination which included gonioscopy, applanation tonometry, disc stereophotography, red-free fundus photography, and visual field testing with automated perimetry. Red-free fundus photographs of the RNFL were taken using a previously described technique [10]. A TRC-50IA (Topcon Inc, Tokyo, Japan) was used. VF tests were performed with the C24-2 program of the Humphrey VF analyzer (Zeiss Inc., San Leandro, CA, USA).

We defined NTG according to the following criteria: (1) intraocular pressure consistently less than 21 mmHg in an eye free from treatment with anti-glaucoma medication, (2) characteristic glaucomatous optic nerve head damage, (3) typical glaucomatous visual field loss, (4) absence of neuroradiologic evidence of optic nerve damage, and (5) open iridocorneal angles and no abnormal chamber angle structure upon gonioscopic examination [10]. Patients with neurological comorbidities by history or abnormal findings on neuroimaging were excluded from the study. We defined the localized RNFL defect as a wedged-shaped defect encroaching upon the optic disc.

The exclusion criteria were as follows: (1) MD (mean deviation) below -14 dB on visual field testing to exclude advanced disease; (2) previous ocular pathology such as retinal disease, cataracts, or a history of ocular surgery; (3) age over 70 years to rule out senile NFL atrophy; and (4) cases where it was difficult to differentiate defects due to the low quality of the red-free photography.

The medical records of 33 patients (42 eyes) with high myopia (spherical equivalent [SE] < −6.0 D), 76 patients (93 eyes) with low to moderate myopia (SE between −6.0 D and −1.0 D), and 54 patients (64 eyes) with emmetropia (SE between −0.5 D and +0.5 D) were reviewed. All patients diagnosed with NTG had RNFL photographs taken. During RNFL analysis, eyes with indefinite localized RNFL defects due to cataracts, or abnormal retinal findings such as diabetic retinopathy, drusen, and epiretinal membranes were excluded. Eyes with diffuse RNFL atrophy based on the OCT (defect of 3 clock hours or more) and red-free photo (which could not be defined as a localized defect) were excluded.

**Figure 1:**
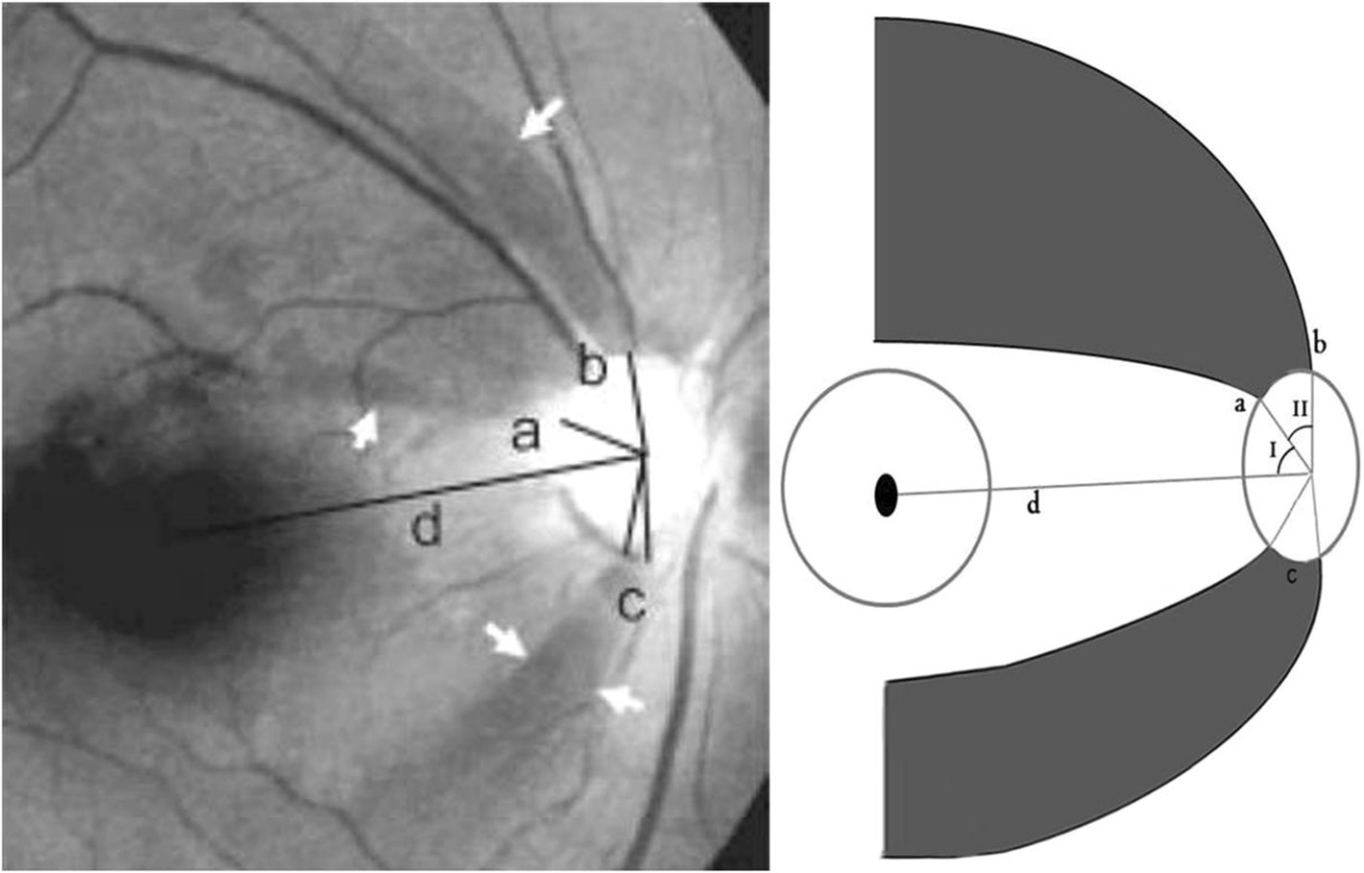
**Parameters for retinal nerve fiber layer (RNFL) photography [**[15]**].** Reference line d extends from the center of the disc to the center of the fovea. Angle I is the minimum angle between the reference line and a line from the disc center to the point of the disc margin nearest the border of the defect. Angle II is the sum (b + c) of the angular widths of the defects.

The parameters of RNFL photography were defined for the evaluation of localized RNFL defects, and are illustrated in Figure 1.

(1) Reference line: a line from the center of the disc to the center of the macula.

(2) Angle I: the minimum angle made by the reference line and a line from the center of the disc to the disc margin where the RNFL defect meets the disc.

(3) Angle II: the width of the RNFL defect at the disc.

Localized RNFL defects with an angle exceeding 120º from the reference line were excluded, since those defects in the nasal RNFL are difficult to identify.

All measurements were obtained by evaluation of the RNFL photographs by an experienced ophthalmologist. To prevent examiner bias, the RNFL evaluation was carried out with the examiner blinded to information regarding the patients’ diagnoses and refractive error. Demographic and clinical data including age, sex, MD, axial length(AL), SE, and intraocular pressure(IOP) were compared across groups. Multiple regression analysis was used to compare the measured parameters of the RNFL defects (angles I and II) between the groups. Chi-squared tests was used to compare categorical variables, and ANCOVA tests adjusted for age and gender with Bonferroni correction were used to compare continuous variables among the 3 groups. P-values <0.05 were considered to be statistically significant.

## Results

**Figure 2:**
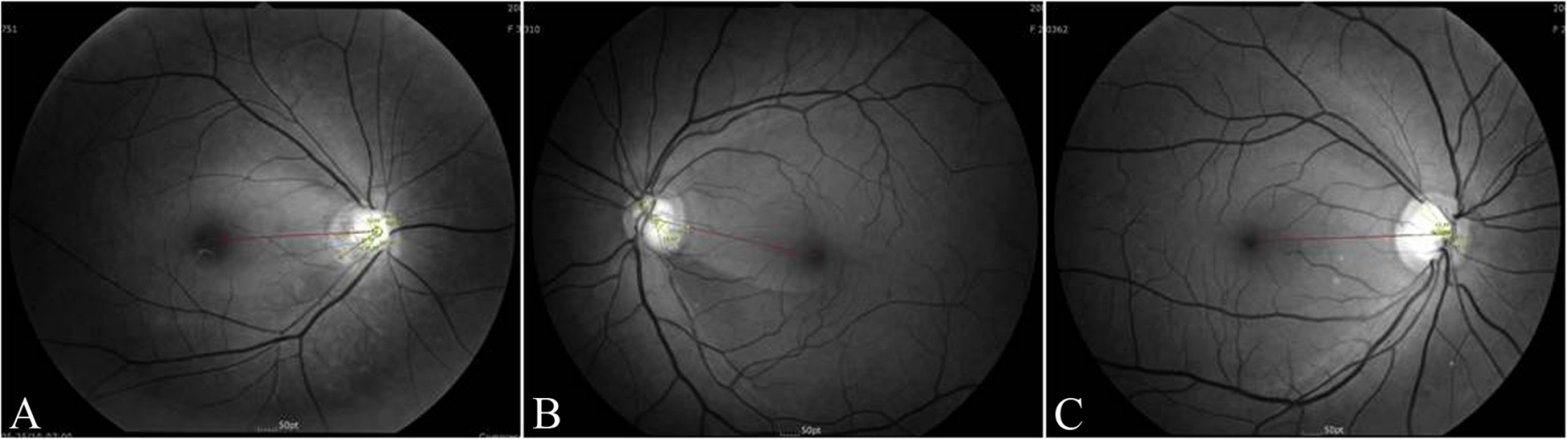
**Typical retinal nerve fiber layer (RNFL) defects in eyes with high myopia (A), low to moderate myopia (B), and emmetropia (C). (A)** The right eye of a 46-year-old male patient with −6.75 diopters of spherical equivalent (angle I = 30.95°, angle II = 59.04°, MD:-3.03). **(B)** The left eye of a 37-year-old female patient with −2.00 diopters of spherical equivalent (angle I = 37.85°, angle II = 25.87°, MD:-1.09). **(C)** The right eye of a 50-year-old female patient with −0.25 diopters of spherical equivalent (angle I = 40.89°, angle II = 15.69 °, MD: -2.97).

The mean patient age was 43.8 ± 11.7 years (22–70 years) in the high myopia group, 48.1 ± 10.5 years (25–70 years) in the low to moderate myopia group, and 55.8 ± 10.2 years (30–70 years) in the emmetropia group. Subjects in the emmetropia group were significantly older than patients in the other two groups (p < 0.001). After adjusting for gender, there was still a significant age difference between groups (p < 0.001). Table 1 shows patient characteristics and a summary of these results. Typical RNFL photographs in eyes with high myopia, low to moderate myopia, and emmetropia are shown in Figure 2.

**Table 1:** Comparison of variables between groups

**Variables**	**Group**	**Mean ± SD**	**Overall p-value**	**Adjusted p-value**
Age	H / M	43.8 ± 11.7 / 48.1 ± 10.5	<0.001^*^	0.099^*^
	H / E	43.8 ± 11.7 / 55.8 ± 10.2		<0.001^*^
	M / E	48.1 ± 10.5 / 55.8 ± 10.2		<0.001^*^
Gender (Male:Female)	H / M	31:11 / 61:33	0.304^†^	
	H / E	31:11 / 34:31	0.026^†^	
	M / E	61:33 / 34:31	0.112^†^	
MD value of Humphrey	H / M	−7.57 ± 4.04 dB / -5.15 ± 3.75 dB	0.001^*^	0.014^*^
	H / E	−7.57 ± 4.04 dB / -4.38 ± 4.02 dB		0.001^*^
	M / E	−5.15 ± 3.75 dB / -4.38 ± 4.02 dB		0.307^*^
Axial length	H / M	26.96 ± 1.05 mm / 24.95 ± 1.14 mm	<0.001^*^	<0.001^*^
	H / E	26.96 ± 1.05 mm / 23.55 ± 0.38 mm		<0.001^*^
	M / E	24.95 ± 1.14 mm / 23.55 ± 0.38 mm		<0.001^*^
Spherical equivalent	H / M	−8.04 ± 1.68D / -3.29 ± 1.65D	<0.001^*^	<0.001^*^
	H / E	−8.04 ± 1.68D / 0.07 ± 0.40D		<0.001^*^
	M / E	−3.29 ± 1.65D / 0.07 ± 0.40D		<0.001^*^
Intraocular pressure	H / M	15.8 ± 3.8 mmHg / 14.7 ± 3.0 mmHg	0.019^*^	0.162
	H / E	15.8 ± 3.8 mmHg / 14.0 ± 2.8 mmHg		0.015
	M / E	14.7 ± 3.0 mmHg / 14.0 ± 2.8 mmHg		0.442

H: high myopia, M: low to moderate myopia, E: emmetropia.

^†^Chi-squared test, ^*^ANCOVA with Bonferroni adjustment for age and gender.

**Figure 3:**
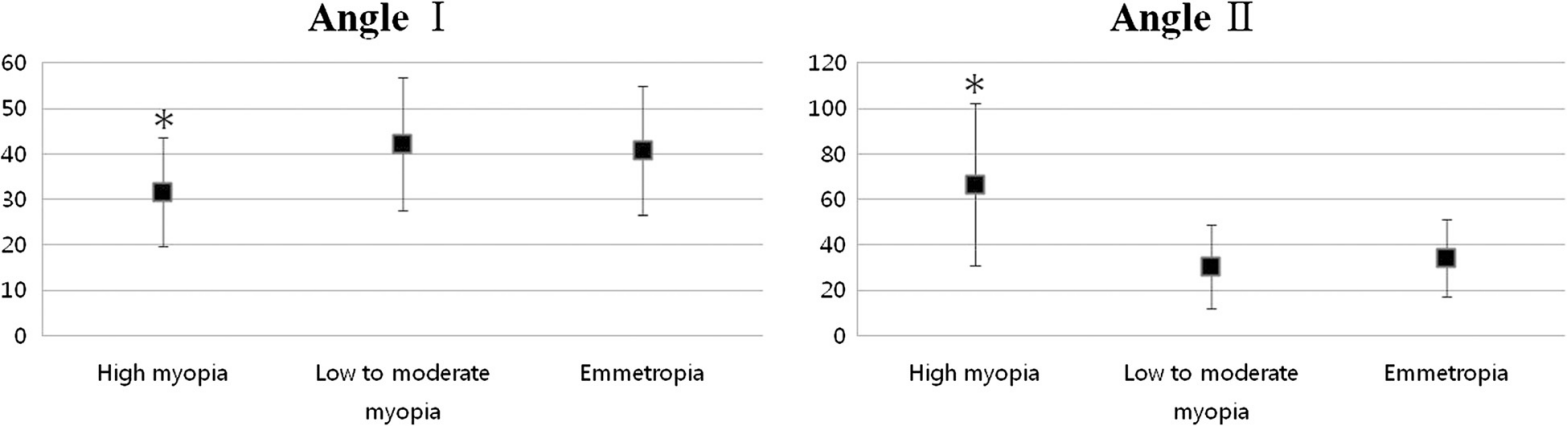
**Average values of angles I and II.** Angle I, the proximity of the defect to the center of the fovea, was significantly smaller in the high myopia group (31.6 ± 11.9° (8.33-74.14)) compared with the low to moderate myopia group (40.8 ± 14.7° (0–77.7)) and emmetropia group (42.2 ± 14.2° (14–75.27)) (p = 0.001 and p < 0.001, respectively). Angle II, the sum of the angular width of the defects, was significantly larger in the high myopia group (66.6 ± 35.9° (12.98-175.64)) compared with the low to moderate myopia group (34.2 ± 18.4° (7.24-91.88)) and emmetropia group (30.4 ± 17.1° (8.37-86.50)) (p < 0.001). ^*^ p-value <0.01 on one-way ANOVA.

The male to female ratios were 3:1 in the high myopia group, 2:1 in the low to moderate myopia group, and 1:1 in the emmetropia group. The high myopia group had a higher male preponderance than the emmetropia group (p = 0.026). MD of Humphrey C24-2 perimetry was significantly reduced in the high myopia group (−7.57 ± 4.04 dB) (95% CI: -8.92, -6.22) compared with the low to moderate myopia group (−5.15 ± 3.75 dB) (95% CI: -5.98, -4.32) and the emmetropia group (−4.38 ± 4.02 dB) (95% CI: -5.47, -3.30) (p = 0.014, p = 0.001). Table 2 shows the comparison values of Angle I and Angle II between groups. Angle I, the proximity of the defect to the center of the fovea, was significantly smaller in the high myopia group (31.6 ± 11.9°) (95% CI: 27.9, 35.3) than in the low to moderate myopia group (40.8 ± 14.7°) (95% CI: 37.8, 43.8) and the emmetropia group (42.2 ± 14.2°) (95% CI: 38.7, 45.7) (p = 0.028 and p = 0.044, respectively). Angle II, the sum of the angular width of the defects, was significantly larger in the high myopia group (66.6 ± 35.9°) (95% CI: 55.4, 77.8) than in the low to moderate myopia group (34.2 ± 18.4°) (95% CI: 30.5, 38.0) and the emmetropia group (30.4 ± 17.1°) (95% CI: 26.2, 34.3) (p < 0.001, p = 0.007) (Figure 3). The differences in angles I and II between the low to moderate myopia group and the emmetropia group were not significant (p > 0.05). In emmetropia and low to moderate myopia, RNFL defects occurred most frequently in the inferior quadrant (30 and 48 cases, respectively), followed by the superior quadrant (21 and 26 cases, respectively), and finally the inferior and superior quadrants (14 and 19 cases, respectively). In contrast, in the high myopia group, simultaneous defects in both quadrants occurred most frequently (27 cases), followed by inferior defects (13 cases) and superior defects (2 cases) (p < 0.001). When both defects were present, the superior defect was wider than the inferior defect in 13 cases (high myopia 7, myopia 3, emmetropia 3) and the inferior defect was wider than the superior defect in 37 cases (high myopia 18, myopia 10, emmetropia 9); there were no significant differences between the groups (p = 0.511).

**Table 2:** Comparisons of angle I and angle II between groups

**Variables**	**Group**	**Mean ± SD**	**Overall p-value**	**Adjusted p-value**
Angle I	H / M	31.6 ± 11.9° / 40.8 ± 14.7°	0.021^*^	0.028^*^
	H / E	31.6 ± 11.9° / 42.2 ± 14.2°		0.044^*^
	M / E	40.8 ± 14.7° / 42.2 ± 14.2°		1.000^*^
Angle II	H / M	66.6 ± 35.9° / 34.2 ± 18.4°	<0.001	<0.001^*^
	H / E	66.6 ± 35.9° / 30.4 ± 17.1°		0.007^*^
	M / E	34.2 ± 18.4° / 30.4 ± 17.1°		1.000^*^

H: high myopia, M: low to moderate myopia, E: emmetropia.

^*^Multivariate regression analysis adjusted for age, gender, spherical equivalent, axial length, and intraocular pressure.

## Discussion

The relationship between myopia and the risk of glaucoma has been extensively investigated [2-4], though it remains controversial. This may be due to difficulties in accurately diagnosing myopia or because abnormal results may occur frequently in patients with high myopia [9]. Few studies have used RNFL photography to study myopic eyes. This is at least in part because RNFL can be obscured by relatively small amounts of pigment in the retinal pigment epithelium (RPE) in patients with myopia. In Caucasian populations, the low amount of RPE pigment may exacerbate this phenomenon. In Asian eyes, visualization of the RNFL in myopic eyes is fairly easy due to the prominence of the RPE pigment.

Our results showed that RNFL defects were larger in size and closer to the macula in the high myopia group compared with the low to moderate myopia and emmetropia groups (Figure 3). Possible mechanisms by which this occurs include: (1) early RNFL defects occur near the macula, (2) RNFL defects progress more quickly, resulting in widening to the macula, or (3) glaucomatous damage may develop at a younger age in patients with high myopia compared to patients with low to moderate myopia or emmetropia, thereby allowing more time for progression. Subjects in the emmetropia group were significantly older than those included in the other 2 groups. The MD values were significantly reduced in the high myopia group compared to the low to moderate myopia and emmetropia groups. Our data indicate that all three mechanisms may play a role in the characteristics of glaucomatous optic nerve damage in myopia. In order to apply the perimetric results, however, prismatic deviation at extra-axial points must be taken into consideration, since some subjects in the high myopia group required use of high-power lenses to correct near vision for the VF examination.

Although the mechanisms linking glaucoma and myopia are poorly understood, some population-based studies have shown an increasing risk of glaucoma with increasing severity of myopia [3,11]. A possible explanation is that in myopic eyes, the optic nerve head may be structurally more susceptible to glaucomatous damage because of weakness in the connective tissue structure and arrangement [12]. Although this has not yet been shown at the histological level, the elongation of the globe in myopic eyes leads to mechanical stretching and thinning of the retina. This elongation of the eyeball is also associated with pathologic changes in the fundus [13,14]. Structural changes associated with myopia, such as a longer axial length, a larger and/or tilted optic disc, and peripapillary atrophy may make the maculopapillary bundle more susceptible to glaucomatous injury [13,14]. The reduction in RNFL thickness with increasing axial length is a potential source of vulnerability in highly myopic eyes. Some studies have suggested that optic nerve damage is more pronounced in highly myopic eyes, and that high myopes have a higher susceptibility to glaucomatous optic neuropathy for any given IOP [15,16].

The relationship between myopia and glaucomatous damage have been evaluated by several groups. Some studies have found that myopic eyes have higher IOPs and severe visual field defects compared with emmetropes [17-19]. Mayama *et al*. reported that higher myopia threatens the remaining lower cecocentral VF, and that it may also negatively affect the quality of vision in advanced stage OAG eyes with high IOPs, but not eyes with low IOPs [18,19]. Despite these data, however, few studies have considered the glaucoma characteristics specific to myopic NTG.

It has been reported that glaucomatous optic nerve damage in highly myopic eyes is more diffuse compared to that of emmetropic eyes [15]. In highly myopic eyes, inferior RNFL defects, atypical fiber defects, and multiple RNFL defects are common [20]. In the early to moderately advanced stages of glaucoma, myopia is positively associated with an abnormality in the cecocentral VF, especially in the lower cecocentral field [18,21]. However, according to the results of this study, in eyes with high myopia RNFL defects occurred frequently in both the superior and inferior quadrants. Additionally, where a defect occurred in a single quadrant, it was often in the inferior quadrant. When defects occurred in both the superior and inferior quadrants, the inferior defects were wider than the superior defects. Because defects were found more frequently in the inferior quadrant than in the superior quadrant, in cases where defects are seen in both quadrants the inferior defect was likely to have occurred first.

In the current study there was a higher male to female ratio in the high myopia group compared to the emmetropia group. While this is an interesting finding, we are unable to draw definitive conclusions from this given the hospital-based, retrospective, cross sectional nature of the study and relatively small number of subjects. To further validate these findings, a longitudinal population-based study is needed. Some studies have failed to demonstrate any gender preference with respect to refractive error and glaucoma [11,22]. However, in the Rotterdam eye study, an association between male gender and high myopia was made with the development of glaucomatous visual field loss [23]. In another study, Wu *et al*. also found that the incidence of moderate-high myopia was related to the presence of glaucoma as well as male gender [24].

There were several limitations to our study. First, because of its retrospective nature, we cannot exclude the possibility of selection bias. Second, we did not adjust the angle for the axial length or refractive error, which may cause differences in magnification. However, we believe that these differences cannot account for the results of this study. Magnification effects should not have altered angular measurements, and the measured arterio-venous ratio in the retina did not change with refractive error [25,26]. However, this does not apply to the cases in which there was irregular magnification, thus we cannot definitely determine whether this impacted our results. Additionally our study was limited by the exclusion of diffuse atrophy as well as a lack of age-matching across groups.

## Conclusion

In summary, this study demonstrates an association between the type of RNFL defect and refractive error. RNFL defects were broad and near the macula in patients with high myopia and NTG. In patients with high myopia, glaucoma is an important consideration because of the high frequency of concurrent defects and VF loss close to fixation. Thus, highly myopic subjects should undergo regular screening for glaucoma starting at a younger age than patients with low myopia or emmetropia. A long-term longitudinal study looking at the degree of myopia and RNFL progression as follow-up covariants in a larger study population is needed to better characterize the relationships between the degree of myopia, IOP, gender, and age in glaucomatous eyes.

## Competing interests

Joon Mo Kim; none, Ki Ho Park; none, Seon Jeong Kim; none, Eun Noh; none, Mi Jeung Kim; none, Dong Myung Kim; none, Tae Woo Kim; none, Joseph Caprioli has received grant support from Pfizer, Inc, Allergan Inc, Alcon, Inc, Research to Prevent Blindness, Inc, and the National Eye Institute, National Institutes of Health, and has received consultant fees from Allergan.

## Authors’ contributions

Literature screening and selection was performed by JMK, KHP, HJJ, EN, MJK and TWK, KHP, TWK, DMK and JC participated in the design of the study. Data collection was done by SJK, HJJ and EN, and JMK and TWK performed the statistical analysis. Preparation of the first draft of the manuscript was done by JMK, critical revision was performed by KHP, SJK, HJJ, EN, MJK, TWK, DMK, and JC and approval of the final version of the manuscript was performed by KHP, SJK, HJJ, EN, MJK, TWK, DMK, and JC. All authors read and approved the final manuscript.

## Pre-publication history

The pre-publication history for this paper can be accessed here:

http://www.biomedcentral.com/1471-2415/13/67/prepub
